# Episiotomy practice and associated factors among mothers who gave birth at public health facilities in Metema district, northwest Ethiopia

**DOI:** 10.1186/s12978-021-01194-9

**Published:** 2021-07-02

**Authors:** Enyew Woretaw, Muluken Teshome, Muluneh Alene

**Affiliations:** 1 Metema Health District, Metema, Ethiopia; 2 Department of Public Health, Debre Markos University, Debre Markos, Ethiopia

**Keywords:** Episiotomy, Factors, Metema, District

## Abstract

**Background:**

Episiotomy is a surgical incision of the perineum to hasten the delivery. There is a scarce of information related to episiotomy practice, and its associated factors, in developing countries, including Ethiopia. Thus, this study was aimed to determine the level of episiotomy practice and to identify its determinants at public health facilities of Metema district, northwest, Ethiopia.

**Methods:**

Institutional-based cross sectional study was conducted among 410 delivered mothers from March 1 to April 30, 2020. We recruited study participants using systematic random sampling technique. Data were entered to Epi data version 3.1 and exported to STATA version 14 for statistical analysis. Stepwise backward elimination was applied for variable selection and model fitness was checked using Hosmer and Lemshows statistics test. Adjusted odds ratio with the corresponding 95% confidence interval was used to declare the significance of variables.

**Results:**

In this study, the magnitude of episiotomy practice was found 44.15% (95% CI 39.32–48.97). Vaginal instrumental delivery (AOR 3.04, 95% CI 1.36–6.78), perineal tear (AOR 3.56, 95% CI 1.68–7.55), age between 25 and 35 (AOR 0.11, 95% CI 0.05–0.25), birth spacing less than 2 years (AOR 4.76, 95% CI 2.31–9.83) and use of oxytocin (AOR 2.73, 95% CI 1.19–6.25) were factors significantly associated with episiotomy practice.

**Conclusions:**

Magnitude of episiotomy practice in this study is higher than the recommended value of World Health Organization (WHO). Instrumental delivery, age, oxytocin, birth spacing and perineal tear were significant factors for episiotomy practice. Thus, specific interventions should be designed to reduce the rate of episiotomy practice.

**Plain English summary**

The routine use of episiotomy practice is not recommended by WHO. A study that compares routine episiotomy with restrictive episiotomy suggests that the latter is associated with less posterior perineal trauma, less need for suturing, and fewer complications related to healing. In addition, though, the rate of episiotomy has been declined in developed countries, still it remains high in less industrialized countries.

The data for this study were taken at public health facilities of Metema district, northwest, Ethiopia. We included a total of 410 delivered mothers. The magnitude of episiotomy practice was found 44%. This result was higher than the recommended value of WHO. The WHO recommends an episiotomy rate of 10% for all normal deliveries.

The result of this study showed that episiotomy practice is common among mothers whose age group are 18–24. In addition, mothers whose labor were assisted by instrumental vaginal delivery are more likely to have episiotomy as compared to those delivered by normal vaginal delivery. Laboring mothers who had used oxytocin were about three times more likely to be exposed for episiotomy than laboring mothers who did not use oxytocin drug. Moreover, episiotomy practice was nearly five times more likely among mothers who had birth spacing of 2 years and less as compared to mothers who had birth spacing of more than 2 years.

## Background

Episiotomy is performed to enlarge the birth outlet in order to facilitate the delivery of the fetus [1]. It is the surgical enlargement of the posterior aspect of the vagina by an incision to the perineum during the last part of the second stage of labor [2]. Even though seven episiotomy types have been identified, only three (midline, mediolateral, and lateral) are routinely used [3]. Types of episiotomy techniques are classified on millimeter distance from the incision point to the posterior fourchette and by angle from the sagittal or parasagittal plane in degrees [4]. Women with midline episiotomy, deep perineal tears occurred in twofold higher compared to women who underwent a medio-lateral episiotomy [5]. The routine use of episiotomy practice is not recommended by World Health Organization (WHO) for women undergoing spontaneous vaginal birth [6]. A meta-analysis of randomized controlled studies that compare routine episiotomy with restrictive episiotomy suggests that the latter is associated with less posterior perineal trauma, less need for suturing, and fewer complications associated with healing [2]. Though the rate of episiotomy has been declined in developed countries, still it remains high in less industrialized countries and East Asia [7].

Routine use of episiotomy originally began by Pomeroy in 1918 and this routine practice was accepted and taught in obstetrics services till 1970s, when the first consistent clinical trials questioning the value of episiotomy were published [8]. Since then many studies, reviews and met-analyses have evidenced that there is no scientific basis for maintaining the routine practice of episiotomy. The procedure is shown to increase intra and post-operative complications, suggesting its practice to be restricted to selected deliveries [9].

Its use has shown also poorer future sexual function, similar pelvic floor muscle strength, and similar urinary incontinence in comparison with women in whom episiotomy is used in a selective manner. Routine use of episiotomy has no evidence on any beneficial effect; on the contrary, there is clear evidence that it may cause harm such as a greater need for surgical repair and a poorer future sexual capability. In view of the available evidence the routine use of episiotomy should be abandoned and episiotomy rates > 30% are not justified. The WHO recommends an episiotomy rate of 10% for all normal deliveries. It is prescribed selectively for women who have past history of lower genital tract surgeries and for women who require assisted vaginal deliveries. For other women in labor, episiotomies may be given on emergency basis when there are presumed imminent perineal tear scar of lower genital track, operative vaginal delivery, macrosomia and tight perineum [10–13].

Strategies for changing practice those were challenging on current practice of episiotomy and on creating social and organizational environments that encourage motivation are more effective in reducing episiotomy rates [14]. A systematic review and meta-analysis that was done on episiotomy recommend that there is an urgent need to explore reasons for and devise programs to reduce the apparent higher rates of episiotomies in low and middle income countries (LMIC) at their medical facilities [14, 15]. Complications of episiotomies include accidental extension into the anal sphincter or rectum, damage to the Bartholin’s gland, unsatisfactory anatomic results such as skin tags, asymmetry or excessive narrowing of the introitus, vaginal prolapse, recto-vaginal fistula, fistula in ano, perineal pain that lasted an average of 5.5 days, oedema, increased blood loss, hematoma, infection and dehiscence [16–23].

Women who had given birth with episiotomy are at risk for psychological trauma, higher frequency of dyspareunia and insufficient lubrication than women who had given birth without episiotomy. Episiotomy may affect women’s sex life during the second year postpartum with more frequent pain and vaginal dryness at intercourse, although the role of episiotomies in the causation of dyspareunia in the long term is not clear [24–26]. Study showed that mean time from delivery to maternal rest and time taken to bond with the infant were significantly longer in the episiotomy groups compared to mothers who delivered without episiotomy procedure. Perineal local infiltration of lidocaine during episiotomy procedure is risk for the newborn for toxication due to maternal perineal nerve block with lidocaine [27, 28].

Even though episiotomy practice is with a decreasing trend in some developed countries, but still statistics revealed that an overall high rates of episiotomy practice around the world. Episiotomy rates ranged from as low as 9.7% (Sweden) to 100% (Taiwan) that include both primiparous and multiparous women. Rates for only primiparas range from 63.3% (South Africa) to 100% (Guatemala), demonstrating that overall greater likelihood of primiparas will undergo episiotomies. In many parts of the world (e.g., Central and South America, South Africa, and Asia)in France population based study showed that episiotomy rate for vaginal deliveries overall significantly decreased from 26.7% in 2007 to 19.9% in 2014 [32, 33].

In our country study showed that, the prevalence of episiotomy at public health institutions of Akaki Kality in Addis Ababa, Axum town, shire town, at saint Paul’s hospital Millennium medical college Addis Ababa and at Mizan Aman General Hospital the prevalence of episiotomy were found to be 35.2%, 41.44%, 35.4%, 65.4%,30.6 respectively [40–44]. Findings at a maternity school in Recife, Pernambuco, Brazil and in a tertiary care centre in Nigeria and in our country at Mizan Aman General Hospital and at Saint Paul’s hospital Millennium Medical College identified that maternal age and place of residence were significant predictors of episiotomy practice [37, 38, 43–45]. Studies in France, Brazil, Iran, Nigeria, Republic of Congo, and in our country studies at Akaki Kality, Axum town, at Saint Paul’s hospital Millennium Medical College and Mizan Aman general hospital identified that Primipara was significant factor for episiotomy practice [33, 37–39, 41, 43–51].

Findings in Israel, Kurdistan region, Republic of Congo, Zimbabwe and in our country at Mizan Aman general Hospital, at Akaki Kality and at Saint Paul’s hospital Millennium Medical College showed that perineal laceration (tear), duration of second stage of labour more than 90 min, ANC follow up history, time of delivery, previous history of episiotomy and known medical diseases were significant predictors for episiotomy practice [43, 44, 46, 47, 49, 52]. Studies in Northeast of Iran, Brazil, Republic of Congo, Israel, Kurdistan region, Zimbabwe and in our country at Mizan Aman general Hospital, at Akaki Kality, at Saint Paul’s hospital Millennium Medical College, at Axum town public health institution and at Jima teaching Hospital identified that birth weight of 4 kg and above, gestational age, presence of meconium, sex the neonate, breech and shoulder presentation and condition of fetal heart rate were significant factors for episiotomy practice [33, 37–39, 41, 43–55].

Studies in Zimbabwe, Brazil, Northeast of Iran, Republic of Congo, Israel, Kurdistan region, and in our country at Mizan Aman general Hospital, at Akaki Kality, at Saint Paul’s hospital Millennium Medical College at Axum town, at Jima teaching Hospital and at Institutions of Shire Town showed that instrumental vaginal delivery especially, use of oxytocin, when doctors attending labor and use of analgesia were predictors of episiotomy practice [33, 37–39, 41–56].

Many countries have recognized that high episiotomy rates are an indicator of high rates of unnecessary obstetric interventions [57]. No question about the rationale for episiotomy, when there is a good indication for its performance. Obstetric perennial trauma is assumed to be a serious health problem for women as well as for their child during their childbirth. Since our country is struggling to improve maternal health, this kind of studies are must to be done in order to improve the well-being and quality of life of women as well [58].

Metema district is one of the hot spot area for HIV transmission in Ethiopia even in 2019 there were 20 mothers who were newly positive at labour and delivery room in this case interventions like episiotomy procedure may increase HIV transmission by 2% compared to normal delivery without episiotomy to the neonate [30, 59, 60]. So, after identifying the magnitude and associated factors of episiotomy practice at Metema District, it is important to give recommendations for this practice. Knowing the magnitude and associated factors of episiotomy with reasons of it in Metema district has a great role in guiding health professionals and health policy makers to identify factors for monitoring episiotomy practice. It also used to apply necessary preventive and appropriate measures to use evidence based restrictive episiotomy practice and to prepare uniform protocols and educational programs to guide episiotomy practice. Hence, the objective of this research was to assess proportions of episiotomy and associated factors among mothers who gave birth at public health facilities in Metema District, Northwest, Ethiopia, 2020.

## Methods

### Study design, area and period

An institutional based cross-sectional study was conducted in Metema District. Metema is one of a hottest District among five Districts in West Gondar Zone which is located 870 km Northwest from Addis Ababa, the capital of Ethiopia, and 315 km far from Bahirdar, a capital of Amhara National Regional State. The most popular crop is sesame. The health facilities in Metema district provided health services to more than 250,000 populations in 2019. Currently, about five health centers and one primary hospital were available in the catchment area of the District. There were more than 5740 deliveries among pregnant mothers in 2019 [61]. This study was conducted from March 1/2020 to April 30/ 2020.

### Sample size and sampling procedures

All women who gave birth vaginally at public health facilities in Metema District during data collection period were included in the study. Mothers who delivered twin and above since it is difficult which child was a factor for episiotomy were excluded.

The required sample size of the study participants for the first objective was determined by using single population proportion formula with basic assumption of 95% confidence interval, 5% margin of error and 41.44%estimated proportion of vaginal delivery with episiotomy at public health institutions of Axum Town, Tigray region, from previous study [41] with 10% of non-response rate was taken to calculate the sample size.

**Fig. 1:**
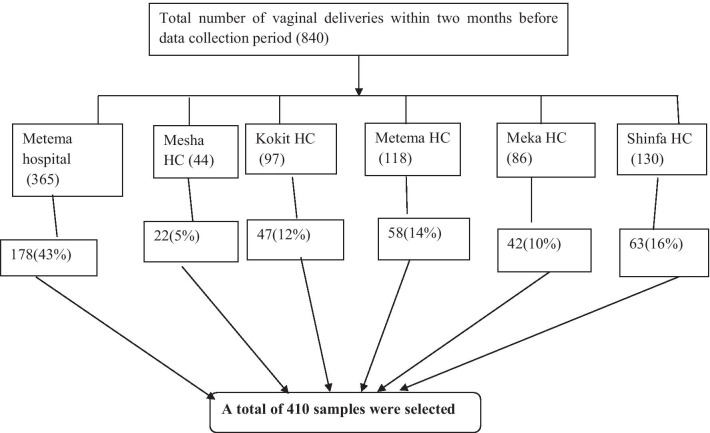
Schematic presentation of sampling procedure for selection of study participants at public health facilitis in Metema district, 2020

In Metema district, there were one primary hospital and five health centers. All the six public health facilities were selected in Metema District and allocated the sample proportionally to all public health facilities based on their previous two months delivery reports. According to pre-assessment done, the two months average total vaginal delivery reports before the start of the study at public health facilities in Metema District were 840. Systematic random sampling technique was used to select the study participants then calculated K for each public health facilities which gave us 2. Then the random number was selected from the numbers [1, 2], using lottery method in this way at Metema primary Hospital, Kokit health center and Mesha health center the selected number was 2 and for others 1 was selected by lottery method and this number was the first study participant to be included in the sample after that every other woman was included in the sample until the total sample size 410 for this study was obtained (Fig. 1).

### Variables

The outcome variable was episiotomy done. The explanatory variables include Socio-demographic and economic factors, clinical and individual maternal factors, clinical and individual child factor and technical factors.

### Operational and term definition

#### Episiotomy done

Is a surgical procedure includes both anterior (defibulation) and posterolateral incisions of the vulva and perineum which is done by health care providers to enlarge the vaginal orifice during delivery.

#### Birth spacing

Interpregnancy interval between live births after the first live birth. Example, short birth interval if the mother gives birth every 2 year or less than 2 years [62].

#### Oxytocin

Is prescribed as a drug for obstetric and gynecological reasons and can help in child birth and considering as a factor in this study if it is given before 3rd stage of labor.

#### Analgesia

A drug for pain management consider if it is given before 3rd stage of labour.

#### Perineal laceration or tear

Is a tear of varying degrees (first to fourth) involving the perineum of women during vaginal birth [63].

### Data collection tool and procedure

The questionnaire was adapted from previously known sources [40, 41]. Income was measured using wealth index by adopting questionnaire from Ethiopian demographic health survey 2016 assuming the income level of participants of rural and urban households differently and it has 15 questions [64]. The questionnaire had five sections with five questions related to socio-demographic characteristics, twelve questions related to clinical and individual maternal factors, six questions related to clinical and individual child factors and seven questions related to technical factors. Data were collected through face to face interview using structured, pre-tested questionnaire and reviewing maternal records (for questions that couldn’t answer by interviewer only) through trained data collectors. The questionnaire was translated from English language to local language Amharic. Seven BSc midwives were recruited to collect data and two BSc midwifes for supervision. The selection criteria of data collectors include interest to participate, being disciplined and punctuality at work. Data were collected in the immediate post-natal period, if conditions unfavorable, it was extended up to 24 h post-delivery.

### Data quality control

One day training was given for 7BSc midwifes on data collection and 2BSc midwifes about supervision during data collection and interviewing approaches. Five percent Pre-test was done in Quara District health facilities before actual data collection was started, and necessary corrections were made accordingly. Data collection completeness and consistency was reviewed and checked by the supervisors and principal investigator at the end of each data collection day. The principal investigator was also closely supervising the activity on daily basis.

### Data processing and analysis

After coding and checking for completeness and consistency, data were entered in to computer using Epi-data version 3.1 and were exported in to stata/SE 14. The data were further recoded, cleaned for missing data outliers before analysis. Data were described using descriptive statistics like frequencies, tables, graph and median with interquartile range. Income level of rural and urban households was separately analyzed and merging them by using principal component analysis and finally wealth index with quintiles was generated. Bivariable analysis using binary logistic regression was done to all independent variables to see their association with the dependent variable. All variables with p value < 0.25 in bivariable analysis were entered into the final multivariable logistic regression model. Then association between dependent and independent variables were assessed using AOR, 95% CI and p-value. Variable with p-value < 0.05 was considered statistically significant. Stepwise backward elimination was applied for variable selection and Hosmer and Lemshow’s statistic test was checked for model fitness of a logistic regression which was Prob > chi^2^ of 0.5559 indicating fitted logistic model since it was greater than p-value of 0.05. Additionally, the model was checked by using receiver operating characteristics which was 0.8851 (area under ROC curve = 0.8851) which indicates excellent model on prediction of accuracy. Lastly Multi-collinearity with Variance inflation factor of each significant variable was checked and it was less than 10 so no multi-collinearity between significant variables.

## Results

This study indicated that the proportion of episiotomy at public health facilities in Metema District was 181(44.15%) among 410 delivered mothers with (95% CI 39.32%, 48.97%).

### Socio-demographic and economic characteristics of respondents

There were a total of 410 mothers who gave birth through vaginal deliveries were interviewed with the response rate of 100%. About, 240 (58.54%) of respondents who gave birth were in the age group of 25–35 years. The median age of the respondents with interquartile range (IQR) were 27 [22–30] years and 234 (57.07%) of respondents were rural residents. Regarding occupation from the total of respondents 259(63.2%) were housewives. Related to the educational status of the respondents, about 201 (49%) were attend primary school and 320 (78.05%) were followers of orthodox religion.

The study finding showed that 98 (23.9%) participants had lowest wealth index and 82(20%) participants had highest wealth index at household level (Table 1).

**Table 1:** Socio-demographic and economic factors of mothers who delivered in Metema District, northwest, Ethiopia, 2020 (n = 410)

Variables	Frequency	Percentage (%)	Variables	Frequency	Percentage (%)
Age group			Religion		
18–24 years	138	33.7	Orthodox	320	78
25–35 years	240	58.5	Muslim	86	21
36–49 years	32	7.8	Protestant	4	1
Educational status			Residence		
No formal education	132	32.2	Urban	176	43
Primary level	201	49	Rural	234	57
Secondary level	64	15.6			
Diploma and above	13	3.2			
Occupation			Wealth index		
House wife	259	63.2	Lowest	98	23.9
Farmer	51	12.4	Second	66	16.1
Merchant	72	17.6	Middle	83	20.2
Daily laborer	8	2	Fourth	81	19.8
Government employee	17	4.2	Highest	82	20
Student	3	0.7			

### Clinical and individual maternal factors

**Fig. 2:**
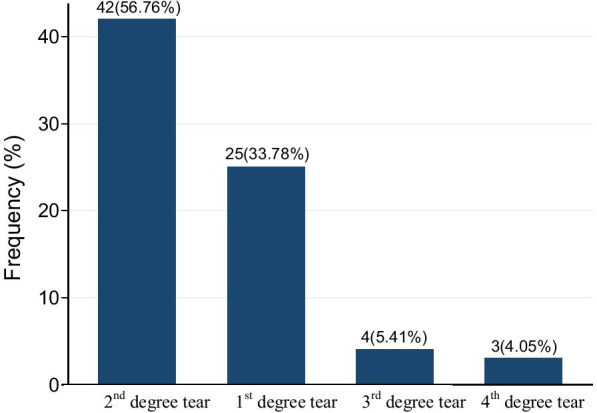
Level of perineal tear among mothers who developed perineal tear during labour at public health facilities in Metema district, northwest, Ethiopia, 2020

From the total of respondents, 345 (84.15%) were had ANC follow up during their pregnancy time and 302(73.7%) of the respondents were multiparous. About, 390(95.1%) of respondents Onset of labor were spontaneous. Among mothers 25(33.78%) had second degree tear during delivery at public health facilities in Metema district (Fig. 2) (Table 2).

**Table 2:** Clinical and individual maternal factors who delivered in Metema district, northwest, Ethiopia, 2020 (n = 410)

Variables	Frequency	Percentage (%)	Variables	Frequency	Percentage (%)
Parity			ANC follow-up history		
Primipara	108	26.3	Yes	345	84.15
Multipara	302	73.7	No	65	15.85
Birth spacing			Duration second stage of labour		
No delivery	108	26.3	≤ 90 min	55	13.41
≤ 2 years	95	23.2	> 90 min	53	12.93
> 2 years	207	50.5			
Medical diseases			Duration of second stage of labour		
Yes	30	7.32	≤ 90 min	55	13.41
No	380	92.68	> 90 min	53	12.93
Time of delivery			Onset of labor		
Day	122	29.8	Spontaneous	390	95.1
Night	288	70.2	Induced	20	4.9
Perineal lacerations					
Yes	74	18.05			
No	336	81.95			

### Clinical and individual child factor

Gestational age of majority of the mothers 339(82.68%) were term at the time of delivery. Regarding fetal condition, 293(71.46%) laboring mothers were with normal condition of fetal heartbeat (Table 3).

**Table 3:** Clinical and individual child factors among mothers who delivered in Metema District, Northwest, Ethiopia, 2020 (n = 410)

Variables	Frequency	Percentage (%)
Sex of the neonate		
Male	242	59.02
Female	168	40.98
Gestational age		
Preterm	16	3.9
Term	339	82.68
Post term	25	6.1
Unknown	30	7.32
Birth weight		
Low	26	6.34
Normal	333	81.22
Macrosomia	51	12.44
Condition of fetal heart beat		
Normal	293	71.46
Bradycardia	63	15.37
Tachycardia	54	13.17
Fetal presentation		
Vertex	359	87.56
Breech	49	11.95
Shoulder presentation	2	0.49
Presence of meconium		
Yes	65	15.85
No	345	84.15

### Technical factors

Three hundred forty six (84.39%) mothers were delivered vaginally, while 64(15.61%) mothers were delivered by instrumental assisted vaginal delivery (Tables 4, 5).

**Table 4:** Technical factors for episiotomy in Metema district, Northwest, Ethiopia, 2020 (n = 410)

Variables	Frequency	Percentage (%)
Use of oxytocin		
Yes	59	14.39
No	351	85.61
Fundal pressure		
Yes	93	22.68
No	317	77.32
Use of analgesia		
Yes	15	3.66
No	395	96.34
Instrumental vaginal delivery		
Yes	64	15.61
No	346	84.39
Birth attendants		
Midwife	386	94.15
HO	5	1.22
Nurse	4	0.98
IESO	9	2.20
Doctor	6	1.46

**Table 5:** Bivariable and multivariable logistic regression analysis of factors associated with practice of episiotomy at public health facilities in Metema district, Northwest, Ethiopia, 2020

Variables	Episiotomy practice		COR (95%CI)	AOR (95%CI)
	Yes (%)	No (%)		
Age group				
18–24 years	110 (26.83)	28 (6.83)	1	1
25–35 years	50 (12.20)	190 (46.34)	0.07 (0.04, .11)	0.11 (0.05, 0.25)
36–49 years	21 ( 5.12)	11 (2.68)	0.49 (.21, 1.12)	0.3 (0.09, 0.99)
ANC follow up history				
Yes	130 (31.71)	215 (52.44)	0.17 (.09,.31)	1.80 (0.78, 4.15
No	51 (12.44)	14 (3.41)	1	1
Vaginal instrumental delivery				
Yes	47 (11.46)	17 (4.15)	4.37 (2.41, 7.93)	3.04 (1.36, 6.78)
No	134 (32.68)	212 (51.71)	1	1
Educational status				
No formal education	89	43	4.66 (1.36, 15.98)	0.85 (0.22, 3.36)
Primary level	69	132	1.18 (0.35, 3.96)	0.28 (0.07, 1.10)
Secondary level	19	45	0.95 (0.26, 3.47)	0.39 (0.09, 1.67)
Diploma and above	4	9	1	1
Oxytocin				
Yes	43 (10.49)	16 (3.90)	4.15 (2.25, 7.65)	2.73 (1.19, 6.25)
No	138 (33.66)	213 (51.95)	1	1
Birth spacing				
No previous delivery history	35 (8.54)	73 (17.80)	9.91 (5.77, 16.99)	1.44 (0.59, 3.53)
Two and less than 2 years	72 (17.56)	23 (5.61)	14.87 (8.23, 26.86)	4.76 (2.31, 9.83)
More than 2 years	171 (41.71)	36 (8.78)	1	1
Perineal tear				
Yes	59 (14.39)	15 (3.66)	6.89 ( 3.75, 12.68)	3.56 (1.68, 7.55)
No	122 (29.76)	214 (52.20)	1	1

### Bivariable and multivariable logistic regression analysis of factors associated with episiotomy practice

The bivariable analysis showed that, maternal age, vaginal instrumental delivery, oxytocin, birth spacing, ANC follow up history, educational status, fetal presentation, duration of second stage of labour, birth weight, onset of labour, and perineal laceration had an association with the episiotomy practice at p-value of 0.25 and these variables were candidate for multivariable logistic regression. But, after multivariable logistic regression analysis, only maternal age, vaginal instrumental delivery, oxytocin, birth spacing and perineal tear were significantly associated with episiotomy at p value less than 0.05 at 95% confidence interval at public health facilities in Metema District, 2020. But other variables (party, time of delivery, Episiotomy previous history, known medical disease, residence, occupation, religion, wealth index, gestational age, fetal heart rate, presence of meconium, sex of the neonate, birth attendants and use of analgesia) had no association with episiotomy practice at p value of 0.25 and were not candidate for multivariable logistic regression.

This study showed that mothers whose age group between 25 and 35 years were 89% less likely to be exposed for episiotomy than mothers whose age group were between 18 and 24 (AOR 0.11, 95% CI 0.05, 0.25). Likewise, mothers whose age group between 36 and 49 were 70% less likely to be exposed for episiotomy than mothers whose age group were between 18 and 24 (AOR 0.3, 95% CI 0.09, 0.99).

Regarding instrumental vaginal delivery, mothers whose labor were assisted by instrumental vaginal delivery were 3.04 times more likely to have episiotomy as compared to those delivered by normal vaginal delivery (AOR 3.04, 95% CI 1.36, 6.78).

Laboring mothers who had used oxytocin were at 2.73 times more likely to be exposed for episiotomy than laboring mothers who did not use oxytocin drug (AOR 2.73, 95% CI 1.19, 6.25).

The odds of episiotomy practice were 4.76 times more likely among mothers who had birth spacing of 2 years and less than 2 years when compared with mothers who had birth spacing of more than 2 years (AOR 4.76, 95% CI 2.31, 9.83).The odds of episiotomy practice were 3.56 times more likely among mothers who had perineal tear during delivery when compared with who had no perineal tear (AOR 3.56, 95% CI 1.68, 7.55).

## Discussion

This study revealed that the magnitude of episiotomy practice is 181(44.15%) with (95% CI 39.32%, 48.97%) which was higher than the recommended value by the WHO [65]. This finding was lower than the previous finding at Saint Paul’s Hospital Millennium Medical College in Addis Ababa which reported that the prevalence of episiotomy was 65.4% [43]. This could be due to the high risk population as the referral hospital deals with referral cases. Study done in Enugu, Southeast, Nigeria and Mulago National Referral Hospital, Uganda and also in Romania the magnitude of episiotomy was 62.1%, 73% and 71.4% respectively [34, 38, 66]. This variation might be explained by the difference in study area, study facilities. However, the magnitude of episiotomy practice found in this study was higher than previous studies conducted at Mizan Aman General Hospital and at public health institutions of Akaki Kality in Addis Ababa, which reported that the prevalence of episiotomy was found to be 30.6%, and 35.2% respectively [44, 46]. This might be due to the previous studies used smaller sample sizes (381, 338 and 310) were study participants respectively. The result of this study also higher than those studies found at King Abdulaziz university hospital in Saudi Arabia and in the United States the proportion of episiotomy were (35%, 24.5%) respectively [67, 68]. This difference might be due to health workers skill gap (gap for skill training) and country policies towards the selective use of episiotomy. The result of this study was comparable to a previous study done at public health institutions of Axum town, north Ethiopia, which reported that, the proportion of episiotomy practice was 41.44% [41]. The reason for those close results might be studying facilities; this study was conducted at one Hospital and health centers which was similar with a study done at Axum town.

The result of this study showed that instrumental vaginal delivery was associated with episiotomy practice. This study showed that mothers whose labor were assisted by instrumental vaginal delivery were 3.04 times more likely to have episiotomy as compared to those delivered by normal vaginal delivery (AOR 3.04, 95% CI 1.36, 6.78), This finding is consistent with studies conducted in Mizan Aman, Axum, Addis Ababa and Nigeria [37, 41, 44, 69]. This might be due to cephalhaematoma, third and fourth degree perineal lacerations during instrumental vaginal delivery procedures.

This study also revealed that laboring mothers who had used oxytocin were at 2.73timesmore likely to be exposed for episiotomy than laboring mothers who did not use oxytocin drug (AOR 2.73, 95% CI 1.19, 6.25). This finding is congruent with studies conducted in Shire town and Northeast of Iran [42, 54]. This might be due to the fact that oxytocin can cause the uterus to contract too strong, which may affect the pattern of baby’s heartbeat.

Moreover, mothers whose age group between 25 and 35 were 89% less likely to be exposed for episiotomy than mothers whose age group were between 18 and 24(AOR 0.11, 95% CI 0.05, 0.25). This result is consistent with the study conducted in Gondar Referral Hospital, Shire Town, Nigeria and Iran [42, 54, 70, 71]. This might be suggests that loosen tenser (tight) musculature of multiparous for increased releasing period of cephalic pole presentation which in turn might not lead to health professionals to do episiotomy.

Likewise birth spacing was significantly associated with episiotomy practice. The odds of episiotomy practice were 4.76 times more likely among mothers who had birth spacing of 2 years and less than 2 years when compared with mothers who had birth spacing of more than 2 years (AOR 4.76, 95% CI 2.31, 9.83). This might be due to the fact that those mothers who had short birth spacing their reproductive organs become weak and delicate this might be a risk for perineal tear.

In this study, the odds of episiotomy practice were 3.56 times more likely among mothers who had perineal tear during delivery when compared with who had no perineal tear (AOR 3.56, 95% CI 1.68, 7.55). This study is consistent with the study conducted in Mizan Aman, Kurdistan region and Israel [44, 49, 52]. This might be due to a reason that episiotomy in such cases is supposed to lower the increased risk of obstetric anal sphincter injuries in subsequent deliveries.

This study had some limitations. It was difficult to know clearly the magnitude of mothers who got episiotomy care due to an indication for episiotomy or due to false initiation of episiotomy.

## Conclusion

The proportion of episiotomy in this study was higher than the recommended value by the WHO (5–10%). Maternal age, vaginal instrumental delivery, oxytocin drug, birth spacing and perineal tear were independent factors for episiotomy practice at public health facilities in Metema District. Therefore reduce unnecessary interventions like giving oxytocin and using instrumental vaginal delivery during normal labor and it is best to know common understanding on perineal tear by preparing morning session on job training.

## Data Availability

All data are available in the manuscript.

## Abbreviations

AIDS: Acquired immune deficiency syndrome
ANC: Antenatal care
AOR: Adjusted odds ratio
APH: Ante-partum hemorrhage
CI: Confidence interval
COR: Crude odds ratio
DM: Diabetes mellitus
HC: Health center
HIV: Human immune deficiency virus
HTN: Hypertension
IESO: Integrated Emergency Surgine Officer
LMIC: Low and Middle Income Countries
PIH: Pregnancy induced hypertension
STATA: Software for Statistics and Data Science
WHO: World Health Organization
